# Reproductive Factors and Non-Hodgkin Lymphoma Risk in the California Teachers Study

**DOI:** 10.1371/journal.pone.0008135

**Published:** 2009-12-02

**Authors:** Jennifer Prescott, Yani Lu, Ellen T. Chang, Jane Sullivan-Halley, Katherine D. Henderson, Christina A. Clarke, Huiyan Ma, Claire Templeman, Dennis Deapen, Leslie Bernstein

**Affiliations:** 1 Program in Molecular and Genetic Epidemiology, Harvard School of Public Health, Boston, Massachusetts, United States of America; 2 Channing Laboratory, Department of Medicine, Brigham and Women's Hospital and Harvard Medical School, Boston, Massachusetts, United States of America; 3 City of Hope National Medical Center, Duarte, California, United States of America; 4 Northern California Cancer Center, Fremont, California, United States of America; 5 Division of Epidemiology, Department of Health Research and Policy, Stanford University School of Medicine, Stanford, California, United States of America; 6 Department of Preventive Medicine, Norris Comprehensive Cancer Center, University of Southern California Keck School of Medicine, Los Angeles, California, United States of America; Health Canada, Canada

## Abstract

**Background:**

Non-Hodgkin lymphoma (NHL) is a malignancy etiologically linked to immunomodulatory exposures and disorders. Endogenous female sex hormones may modify immune function and influence NHL risk. Few studies have examined associations between reproductive factors, which can serve as surrogates for such hormonal exposures, and NHL risk by subtype.

**Methodology/Principal Findings:**

Women in the California Teachers Study cohort provided detailed data in 1995–1996 on reproductive history. Follow-up through 2007 identified 574 women with incident B-cell NHL. Hazard rate ratios (RR) and 95% confidence intervals (CI) were estimated using Cox proportional hazards models to assess associations between reproductive factors and all B-cell NHL combined, diffuse large B-cell lymphomas, follicular lymphomas, and B-cell chronic lymphocytic leukemias/small lymphocytic lymphomas. Pregnancy was marginally associated with lower risk of B-cell NHL (RR = 0.84, 95% CI = 0.68–1.04). Much of the reduction in risk was observed after one full-term pregnancy relative to nulligravid women (RR = 0.75, 95% CI = 0.54–1.06; P for trend <0.01), particularly for diffuse large B-cell lymphomas (P for trend = 0.13), but not among women who had only incomplete pregnancies. Age at first full-term pregnancy was marginally inversely associated with B-cell NHL risk overall (P for trend = 0.08) and for diffuse large B-cell lymphomas (P for trend = 0.056). Breast feeding was not associated with B-cell NHL risk overall or by subtype.

**Conclusions:**

Full-term pregnancy and early age at first full-term pregnancy account for most of the observed reduction in B-cell NHL risk associated with gravidity. Pregnancy-related hormonal exposures, including prolonged and high-level exposure to progesterone during a full-term pregnancy may inhibit development of B-cell NHL.

## Introduction

Non-Hodgkin lymphomas (NHLs) are a heterogeneous group of lymphoid malignancies. In the US, NHL incidence rates have risen steadily since the early 1940s [1], such that NHL is now the fifth most common cancer among US men and women [2]. Although immunodeficiency, autoimmunity, and certain viral infections have been associated with increased risk of some NHLs, the risk factors responsible for the development of most NHLs remain elusive [3], [4]. The observation that men have consistently higher incidence rates of NHL than women [5], coupled with biological evidence that sex steroid hormones modulate the immune system [6], suggests a potential influence of endogenously produced female hormones on lymphomagenesis. Case-control [7]–[12] and cohort studies [13]–[16] that have used various menstrual and reproductive characteristics to assess the relationship between endogenous estrogen exposure and NHL risk in women have produced inconsistent results.

Different NHL histologic subtypes are thought to arise from the clonal expansion of malignant cells during progressive stages of lymphocyte differentiation [17]. The diverse incidence patterns observed for distinct histologic subtypes of NHL suggest that subtypes differ etiologically [5], but few studies [11], [12], [16] have examined menstrual and reproductive risk factors for NHL subtypes. Zhang et al [11] showed an inverse association with number of pregnancies, which was particularly pronounced for diffuse NHL, as defined by the Working Formulation [18]. Similarly, Lee et al [12] found that women with four or more pregnancies had decreased risk of diffuse large-cell lymphoma (defined by the REAL classification [19], and not limited to B-cell NHL) compared to women who had never been pregnant. In contrast to these case-control studies, a recent cohort study [16] reported no association between parity and overall NHL or diffuse large B-cell lymphoma, classified according to the World Health Organization [20] and the International Lymphoma Epidemiology Consortium (InterLymph) guidelines [21]. Morton et al. [16] observed a non-significant reduction in NHL risk associated with older age at menarche that appeared restricted to follicular lymphoma risk. We used comparatively large sample sizes of the common NHL histologic subtypes from the prospective California Teachers Study to address whether menstrual and reproductive factors influence risk of common B-cell NHL subtypes.

## Materials and Methods

### Study Population

The California Teachers Study is a prospective cohort comprising 133,479 female public school professionals recruited through the California State Teachers Retirement System. In 1995-1996, participants completed a detailed self-administered questionnaire, which gathered information on menstrual and reproductive factors, hormone use, medical history, and lifestyle factors. A detailed description of this cohort has been published elsewhere [22].

For this analysis, we excluded sequentially women who were not California residents at the time the baseline questionnaire was completed (n = 8,867), had limited their participation to breast cancer research (n = 18), had a prior history of a hematologic malignancy (n = 536) or whose history of cancer was unknown (n = 663), were 85 years of age or older at baseline (n = 2179), were pregnant at the time they completed the questionnaire (n = 157), or had never had a first menstrual period (n = 55). Our final analytic cohort consisted of 121,004 women.

### Ethics Statement

Completion of the self-administered written questionnaire was considered to imply informed consent. A small subset of women restricted use of their information to breast cancer research and have been excluded from this analysis. Use of human subjects in this study was approved by the Institutional Review Boards at the City of Hope, University of Southern California, Northern California Cancer Center and University of California Irvine, and by the Committee for the Protection of Human Subjects, California Health and Human Services Agency.

### Case Ascertainment and Follow-Up

California law requires the reporting of all new cancer diagnoses to the statewide, population-based California Cancer Registry [23]. As a result, the registry receives information on over 99% of all cancers diagnosed in California (www.ccrcal.org). Annual linkages to the California Cancer Registry identified 574 incident cases of B-cell NHL (International Classification of Diseases for Oncology, third edition [ICD-O-3] morphology codes: 9590, 9591, 9670–9675, 9678–9699, 9727, 9823, 9832, 9835, 9836) in the analytic cohort through December 31, 2007. Among the cases identified were 155 diffuse large B-cell lymphomas (9678–9680, 9684), 121 follicular lymphomas (9690–9698), and 124 B-cell chronic lymphocytic leukemias/small lymphocytic lymphomas (CLL/SLL; 9670, 9823).

Follow-up for each participant began on the date the baseline questionnaire was completed and continued until the first of the following outcomes: a first diagnosis of a hematologic malignancy, a permanent move outside of California, death, or the end of the follow-up period (December 31, 2007). Women who were diagnosed during follow up with T-cell NHL, multiple myeloma, Hodgkin lymphoma or leukemia other than chronic lymphocytic and prolymphocytic leukemias were censored on their dates of diagnosis. The status of California residence was monitored through linkages with the California Department of Motor Vehicles and the U.S. Postal Service national change of address database, as well as through address corrections received from the post office and change-of-address postcards submitted by participants. Information on the date and cause of death were obtained from linkages to state and national mortality files [22].

### Exposure Assessment

At baseline, participants provided detailed information on age at menarche; whether they had ever been pregnant (yes or no); age at and outcome of each pregnancy (induced abortion, miscarriage, tubal pregnancy, stillbirth or live birth); if they had breastfed, total duration of breast feeding; and menopausal status. In this analysis, full-term pregnancy combined live births and stillbirths. When assessing associations with number of full-term pregnancies, we used women who had never been pregnant (nulligravid women) as our reference group, and grouped together women who had had an induced abortion, miscarriage, or tubal pregnancy, but no live birth or stillbirth. We also examined the effects of age at first full-term pregnancy and duration of breast feeding among parous women.

Perimenopausal women were defined as women whose menstrual periods had stopped within 6 months of baseline. Women were considered postmenopausal if their menstrual periods had stopped (due to natural menopause or bilateral oophorectomy) more than 6 months before completing the baseline questionnaire or they were 56 years of age or older and not considered premenopausal or perimenopausal. Postmenopausal women were subdivided according to their use of hormone therapy (HT) into women who had never used HT for menopausal symptoms and those who had used HT (estrogen alone or combined estrogen and progestin therapy) for menopausal symptoms.

### Statistical Analyses

We used multivariable Cox proportional hazards regression models to investigate associations between reproductive factors and all B-cell NHL combined as well as for the 3 most common NHL subtypes. Hazard rate ratios (RR) and 95% confidence intervals (CI) were estimated using age in days from baseline until the end of follow-up as the time scale in the Cox regression models. All models were stratified by age in years at baseline.

We assessed race (white, non-white, or unknown), residential area-level socioeconomic status (quartiles of California statewide distribution [24] or unknown), first-degree family history of lymphoma (no, yes, or unknown), body mass index (BMI) at baseline (<20 kg/m^2^, 20–24.9 kg/m^2^, 25–29.9 kg/m^2^, ≥30 kg/m^2^, or unknown), alcohol consumption in the year before joining the cohort (none, <15 g/day, ≥15 g/day, or unknown), smoking status at baseline (never, former, current, or unknown), oral contraceptive use (no, yes, or unknown), menopausal and HT use status (premenopausal, peri-menopausal, postmenopausal with no HT use, postmenopausal with any HT use, postmenopausal with unknown HT use, or unknown menopausal/HT use status), and any prior diagnosis of diabetes (no or yes) as potential confounders of the relationship between reproductive variables and risk of B-cell NHL. None of these factors altered risk estimates by as much as 5% for overall B-cell NHL, and therefore none were included in the final models. We included age at menarche in years (<12, 12, 13+, or unknown) as a covariate in all regression models. Age at and number of pregnancies overall, or full-term pregnancies, were mutually adjusted for one another.

To test for trend, ordinal variables representing the median within each category were included in the regression models for number of full-term pregnancies, months of breast feeding, and age at first full-term pregnancy. Two-sided P values are reported for tests for trend. We did not adjust P values for multiple comparisons. All statistical analyses were performed using SAS version 9.1 (SAS Institute Inc, Cary, NC).

## Results

The average length of follow-up for women in the analytic cohort was 11 years. The mean ages (±SD) at diagnosis were 69.5±11.6 years (range, 33–93) for all B-cell NHL combined, 69.3±11.7 years (range, 33–92) for diffuse large B-cell lymphoma, 66.3±12.4 years (range, 35–90) for follicular lymphoma, and 72.1±9.7 (range, 48–92) for CLL/SLL.

Table 1 provides the age-adjusted distribution of several participant characteristics according to number of full-term pregnancies. At baseline, 56.6% of participants reported at least 2 full-term pregnancies. Women who had at least 2 full-term pregnancies were more likely to be white and older, but less likely to be current smokers at baseline or to have consumed at least 15 grams/day of alcohol in the year prior to joining the cohort. Women who had at least 3 full-term pregnancies were more likely to be postmenopausal.

**Table 1:** Selected baseline characteristics of 121,004 eligible women in the California Teachers Study by number of full-term pregnancies at baseline.

Characteristic	Subcategory	N (total)	Number of full-term pregnancies (%)				
			0	1	2	3+	Unknown
Total		121004	26.1	15.4	32.2	24.4	1.9
Age categories (years)	<35	12596	56.0	21.6	16.4	4.3	1.8
	35–44	21417	28.8	16.9	36.9	16.2	1.2
	45–54	36455	23.9	17.0	39.6	18.2	1.3
	55–64	23464	19.5	11.8	31.6	35.4	1.8
	65–74	17790	17.5	10.5	26.2	43.1	2.8
	75–84	9282	20.5	16.3	27.4	31.2	4.6
Race	Non-Hispanic white	104636	24.1	15.2	35.5	23.7	1.5
	Other races/ethnicities	15373	27.1	17.9	30.4	22.3	2.3
	Unknown race/ethnicity	995	22.0	15.4	29.0	27.0	6.7
First-degree family history of lymphoma	No	114072	24.5	15.5	34.9	23.5	1.6
	Yes	3177	21.8	15.2	35.7	25.9	1.5
	Unknown/Adopted	3755	26.5	17.6	31.4	21.9	2.7
Socioeconomic status	Below statewide median	26037	25.3	15.7	31.3	26.0	1.7
	Above statewide median	93422	24.2	15.5	35.8	22.9	1.6
	Unknown	1545	25.5	15.6	32.3	24.7	1.9
Diagnosed with diabetes	No	117557	24.5	15.5	35.0	23.4	1.6
	Yes	3447	23.7	16.1	29.4	29.1	1.9
Alcohol consumption (grams/day)	None	38533	23.1	15.5	33.9	25.8	1.7
	<15	56877	24.1	15.7	36.3	22.5	1.5
	≥15	19285	28.0	15.2	33.0	22.1	1.6
	Unknown	6309	25.2	16.5	32.6	23.1	2.6
Smoking status	Never	79453	24.2	15.2	35.5	23.6	1.6
	Former	34652	24.3	16.2	34.3	23.7	1.6
	Current	6176	29.0	16.9	30.5	21.8	1.8
	Unknown	723	25.1	17.1	31.8	22.5	3.5
Body mass index (kg/m^2^)	16–19.9	12614	29.3	17.1	33.6	18.5	1.5
	20–24.9	58086	24.2	15.9	36.1	22.3	1.5
	25–29.9	29061	22.1	14.4	35.0	26.9	1.6
	30–54.9	16399	26.4	15.1	32.0	25.0	1.5
	Unknown	4844	22.3	15.6	31.5	26.7	3.9
Oral contraceptive use	No	38485	31.0	11.8	25.5	30.2	1.3
	Yes	79023	22.3	17.0	38.7	21.6	0.4
	Unknown	3496	21.1	11.3	17.8	12.1	37.7
Menopausal status/hormone therapy (HT) use	Premenopausal	47781	28.2	17.9	36.9	16.2	0.8
	Perimenopausal	2514	25.7	16.8	38.6	18.4	0.5
	Postmenopausal, no HT use	14056	24.1	13.6	28.2	32.0	2.1
	Postmenopausal, HT use	46588	21.3	13.8	33.7	29.5	1.8
	Postmenopausal, unknown HT use	4646	21.1	13.8	31.0	30.8	3.3
	Unknown menopuasal/HT use status	5419	21.4	14.8	39.5	19.8	4.5

Gravid women (women who had been pregnant) had lower risk of overall B-cell NHL than nulligravid women (women who had never been pregnant) (RR = 0.84, 95% CI = 0.68–1.04; **Table 2**). Full-term pregnancy appeared to account for most of the reduction in risk, since we saw no association with NHL risk among women who had been pregnant but never carried to full-term, compared with nulligravid women (RR = 1.12, 95% CI = 0.74–1.70). Among parous women (women who gave birth), the reduced risk was relatively constant with increasing number of full-term pregnancies compared to nulligravid women (P for trend <0.01). The inverse association with number of full-term pregnancies was strongest for diffuse large B-cell lymphoma, although some indication of reduced risk was also observed among the other histologic subtypes examined.

**Table 2:** Relative risk estimates and 95% confidence intervals for the association between menstrual and reproductive factors and B-cell NHL risk overall and by histologic subtype in the California Teachers Study.

			All cases		Diffuse large B-cell		Follicular		CLL/SLL	
Baseline variable	Subcategory	Person-years	Cases(574)	Multivariate RR (95% CI)	Cases(155)	RR(95% CI)	Cases(121)	RR (95% CI)	Cases(124)	RR (95% CI)
Age at menarche§	<12 years	297699	134	1.00	40	1.00	28	1.00	27	1.00
	12 years	359691	160	0.98 (0.78–1.23)	39	0.80 (0.51–1.24)	34	1.02 (0.62–1.68)	34	1.02 (0.62–1.69)
	>12 years	653476	270	0.86 (0.69–1.05)	74	0.78 (0.53–1.15)	56	0.88 (0.56–1.39)	60	0.93 (0.59–1.46)
	Unknown	17763	10		2		3		3	
	P trend			0.10		0.25		0.51		0.70
Ever pregnant†	No	266779	109	1.00	29	1.00	24	1.00	21	1.00
	Yes	1046397	455	0.84 (0.68–1.04)	124	0.86 (0.57–1.29)	94	0.82 (0.52–1.29)	100	0.94 (0.58–1.50)
	Unknown	15453	10		2		3		3	
Number of FTP† ‡	Never pregnant	266779	109	1.00	29	1.00	24	1.00	21	1.00
	No FTP	77654	28	1.12 (0.74–1.70)	6	0.90 (0.37–2.18)	8	1.34 (0.60–2.99)	1	0.24 (0.03–1.75)
	1	204480	78	0.75 (0.54–1.06)	25	0.76 (0.40–1.45)	18	0.69 (0.33–1.45)	15	0.77 (0.36–1.64)
	2	433421	170	0.72 (0.54–0.95)	44	0.64 (0.37–1.12)	31	0.56 (0.30–1.06)	46	0.98 (0.54–1.78)
	3+	322158	176	0.71 (0.55–0.93)	47	0.68 (0.41–1.14)	37	0.73 (0.41–1.30)	38	0.71 (0.39–1.27)
	Unknown	24136	13		4		3		3	
	P trend			<0.01		0.13		0.21		0.41
Number of pregnancies† ‡	Never pregnant	266779	109	1.00	29	1.00	24	1.00	21	1.00
	1	182735	79	0.94 (0.68–1.31)	24	0.97 (0.521.83)	17	0.82 (0.40–1.68)	14	0.89 (0.42–1.89)
	2	351934	141	0.79 (0.60–1.05)	37	0.74 (0.43–1.29)	26	0.67 (0.36–1.25)	35	0.97 (0.53–1.79)
	3	260357	109	0.73 (0.55–0.98)	29	0.70 (0.40–1.23)	24	0.79 (0.43–1.46)	24	0.77 (0.41–1.46)
	4+	242121	123	0.78 (0.59–1.02)	32	0.74 (0.43–1.26)	27	0.87 (0.49–1.56)	27	0.80 (0.44–1.46)
	Unknown	24704	13		4		3		3	
	P trend			0.01		0.10		0.54		0.23
Age at first full-term pregnancy† ‡ ^||^	<25 years	344503	151	1.00	38	1.00	30	1.00	34	1.00
	25–29 years	391649	185	1.27 (1.02–1.58)	46	1.26 (0.81–1.96)	34	1.51 (0.87–2.61)	49	1.47 (0.94–2.30)
	≥30 years	223888	88	1.21 (0.91–1.61)	32	1.73 (1.04–2.90)	22	1.58 (0.80–3.10)	16	0.99 (0.53–1.86)
	P trend			0.08		0.056		0.11		0.69
Duration of breast feeding† ^||^	None	212897	125	1.00	34	1.00	28	1.00	31	1.00
	<6 months	231266	108	0.94 (0.73–1.22)	32	1.01 (0.62–1.64)	17	0.65 (0.35–1.18)	24	0.87 (0.51–1.48)
	6–11 months	180927	69	0.93 (0.69–1.25)	23	1.13 (0.66–1.92)	15	0.84 (0.45–1.58)	12	0.70 (0.36–1.37)
	12+ months	330667	118	1.02 (0.79–1.32)	26	0.81 (0.48–1.36)	24	0.82 (0.47–1.44)	31	1.23 (0.74–2.03)
	P trend			0.68		0.56		0.79		0.50

FTP: Full-term pregnancy RR = relative risk; CI = confidence interval; CLL = chronic lymphocytic leukemia; SLL = small lymphocytic lymphoma.

† Adjusted for age at menarche.

‡ Number of pregnancies/full-term pregnancies and age at first pregnancy/first full-term pregnancy were mutually adjusted for each other.

§ Adjusted for ever been pregnant.

|| Limited to women with at least one full-term pregnancy.

Among parous women, those who had a first full-term pregnancy at age 25 years or older were at higher risk of overall B-cell NHL than those who had a first full-term pregnancy before age 25 (P for trend with increasing age at first full-term pregnancy = 0.08). This association was most apparent for diffuse large B-cell lymphoma (P for trend = 0.056), but also for follicular lymphomas (P for trend = 0.11). Among parous women, breastfeeding was not associated with risk of overall B-cell NHL or any of the histologic subtypes. Although not statistically significant, some suggestion of reduced risk was observed between later age at menarche and overall B-cell NHL; the RR estimate was lowest for diffuse large B-cell lymphoma.

Secondary analyses restricted to women whose reproductive history at baseline was likely to remain unchanged throughout the cohort follow-up period (i.e., ≥45 years of age at baseline) yielded similar results (data not shown).

## Discussion

Because women generally have lower incidence rates of most NHL subtypes than men [5] and sex hormones have profound effects on the immune system [6], we hypothesized that endogenous estrogen exposure would confer a reduced risk of B-cell NHL. Inconsistencies reported in the literature may be the result of combining histologic subtypes which have heterogeneous clinical features and incidence patterns and thus are likely to possess distinct sets of risk factors [5]. Although we observed about a 30% decreased risk of B-cell NHL overall associated with full-term pregnancies, which induces prolonged exposure to increased circulating estrogen levels [25], [26], our study results are not consistent with a major role for endogenous estrogens in the etiology of B-cell NHL. If our hypothesis was correct, we would expect that late age at menarche would increase the risk of B-cell NHL, as a late age at menarche is associated with lower estrogen levels [27], [28]. Instead, later age at menarche was non-significantly associated with reduced risk of B-cell NHL.

Two previous studies have looked at the association between age at menarche and risk of NHL as a single entity [11], [14], whereas two other studies examined the association with risk by NHL subtypes [12], [16]. No association was observed in the prospective Iowa Women's Health Study [14] or in a population-based case-control study in the San Francisco Bay Area of California [12]. The NIH-AARP Diet and Health Study Cohort observed a non-significant reduction in follicular lymphoma risk among women who had their first menstrual period at age 15 or older compared to women who began menarche at age 12 or younger (RR = 0.58, 95% CI = 0.25–1.35) [12], [16]. On the other hand, a population-based case-control study in Connecticut [11] found an increased risk among women with age at menarche at 15 years or older, compared with those with age at menarche younger than 12 years (OR = 1.5, 95% CI = 1.0–2.2), with no apparent dose-response trend. We observed a non-significant reduced risk of overall B-cell NHL associated with later age at menarche (>12 years vs <12 years RR = 0.86, 95% CI = 0.69–1.05), which was most pronounced for diffuse large B-cell lymphoma. Given the inconsistency in the literature and lack of statistically significant results, the evidence suggests age at menarche does not serve as a surrogate for risk of B-cell NHL or any of the common NHL histologic subtypes.

The association between pregnancy and overall NHL risk has also been inconsistent in previous studies. A population-based case-control study in Los Angeles County, California [10], and the Iowa Women's Health Study [14] found some indication of an increased risk of NHL among parous women, although the relative risk estimates and trends were not statistically significant. Three studies found no association between pregnancy and overall NHL risk [8], [9], [16], whereas the Connecticut case-control study found a statistically significant inverse association between number of full-term births and overall NHL risk [11]. NHL risk was reduced among women with at least 4 full-term pregnancies compared to women who had never been pregnant (OR = 0.6, 95% CI = 0.4–0.9).

Inconsistencies persist when parity is examined by NHL histologic subtypes. A prospective, registry-based study [8] observed a non-significant inverse trend between parity and CLL risk, but did not examine other NHL subtypes separately. Lee et al. [12] reported a statistically significant reduced risk for diffuse large-cell lymphoma, but not for follicular lymphoma. Whereas Zhang et al. [11] observed a non-significant reduced risk of follicular lymphoma and a marginally significant reduced risk of diffuse large B-cell lymphoma with number of full-term pregnancies. Morton et al. [8], [9], [16] did not observe an association of parity with any NHL subtype. In our cohort study, the inverse association with number of full-term pregnancies was most pronounced for diffuse large B-cell lymphoma risk. While the lack of a trend among parous women in our data could result from a threshold effect, the overall inconsistency in the literature also suggests our observations may be a chance finding.

Most studies did not find a statistically significant association between age at first full-term pregnancy and NHL risk overall, although some indicated that the highest-risk group was women who had a first full-term pregnancy between ages 25 to 30 [8], [10]–[12], [14]. Late age at first full-term pregnancy was a risk factor for overall NHL in a hospital-based case-control study in Sweden [7]. By contrast, in a registry-based cohort study in Denmark, age at first birth was inversely associated with NHL risk, as women whose first birth occurred between ages 15 and 19 years had the highest risk [15]. We found a marginally statistically significant positive association between age at first full-term pregnancy and risk of overall B-cell NHL and diffuse large B-cell lymphoma, with some suggestion for increased follicular lymphoma risk, but not CLL/SLL. Although Lee et al. [12] also investigated different NHL subtypes, they used nulliparous women as reference group instead of restricting the analysis to parous women, making our results non-comparable. Nevertheless, they found that age at first birth had no association with overall NHL risk or follicular lymphoma, but women who had their first child after age 28 had a statistically significant decreased risk of diffuse large-cell lymphoma.

Consistent with findings from a previous case-control study [10], breast feeding did not influence NHL risk among parous women in our study. The Iowa Women's Health Study [14], the only other cohort study to assess the effect of breast feeding, found that parous women who had breast fed at least 3 children had a reduced risk of NHL when considered as a single entity.

If our observed inverse association between pregnancy and B-cell NHL risk in our study is true, it prompts the question of how pregnancy might influence NHL development. To promote proper implantation and protect the fetal allograft from rejection during pregnancy, the maternal immunologic state shifts from a predominantly cell-mediated response to a humoral response (reviewed in [29]). One potential mechanism for this shift may involve the steady increase in circulating progesterone levels that occurs throughout pregnancy [30]. Current evidence suggests that once a sufficient level is reached, progesterone stimulates the release of progesterone-induced blocking factor (PIBF). Progesterone and PIBF inhibit T-helper 1 (Th1) cells from producing pro-inflammatory cytokines that activate cell-mediated responses (e.g., interferon-gamma, tumor necrosis factor-alpha, and interleukin 2 (IL-2) and stimulate Th2 cell secretion of cytokines that promote B-cell antibody production (e.g., IL-4, IL-5, IL-6, and IL-10), some of which may be anti-inflammatory. IL-4 further induces Th2 ctyokine synthesis and inhibits Th1 cytokine secretion, reinforcing this shift toward humoral immunity [29], [31].

High progesterone levels, alone or in conjunction with high estrogen exposure, may provide an alternative explanation for the putative protective effect of pregnancy on B-cell NHL risk. The effect may be mediated directly by inducing the differentiation of B-lymphocytes to produce antibodies, or indirectly by promoting a systemic anti-inflammatory environment. Future studies examining cytokine profiles and subsequent B-cell NHL risk among women according to type and dose of exogenous hormone use (i.e., oral contraceptives and postmenopausal hormones) may help tease out the potential effects of estrogen and progesterone.

Our study benefits from its large size, prospective design, and virtually complete case ascertainment through the statewide, population-based California Cancer Registry. Previous population-based case-control studies were unable to interview a large proportion of eligible cases because of severe illness or death [10], [32], [33]. The prospective nature of our study eliminates the potential for survival bias, particularly among patients diagnosed with more aggressive histologic subtypes, such as diffuse large B-cell lymphoma [17]. Over an average 11 years of follow-up, a modest number of cases were available (N = 574) for analyses of overall B-cell NHL risk. Although we examined the largest number of common histologic subtypes for a prospective study compared to prior literature, our samples sizes were still limited for NHL histologic subtype analyses, and we lacked sufficient power to examine associations with less common subtypes. We observed some heterogeneity among the histologic subtypes, highlighting the need to analyze the NHL subtypes as distinct diseases. However, some of the apparent heterogeneity may have been due to the lack of power to detect associations with certain subtypes. Like other cohort studies, ours may have been subject to some exposure misclassification among women whose reproductive history changed after the baseline questionnaire. However, our results did not differ when we repeated analyses including only participants ≥45 years of age at baseline whose reproductive history was unlikely to change. A final consideration regards the limited generalizability of our results, as the CTS does not include women in the lowest socioeconomic status category and is composed primarily of white, non-Hispanic participants (86% of the cohort). Although adjusting for race did not alter our risk estimates by as much as 5%, incidence rates for histologic subtypes differ substantially by race [5], and thus necessitate further study of reproductive factors in B-cell NHL risk among minority populations.

In summary, our results do not support a simple protective role for endogenous estrogen in B-cell NHL development, but if true, may instead suggest a relevant immunomodulatory effect of high progesterone concentrations that are encountered during late pregnancy. Histologic subtype-specific associations differ, supporting the notion that B-cell NHL histologic subtypes are indeed etiologically and clinically distinct diseases. Future prospective studies with long follow-up periods are needed to accrue sufficient sample sizes to examine risk factor associations with risk of individual B-cell NHL histologic subtypes, and perhaps with T-cell NHL. Studies assessing detailed exogenous hormone use could also help tease out whether estrogen and progesterone contribute to B-cell NHL risk, and perhaps help explain the gender disparity in NHL incidence.

## References

[1] Zheng T, Mayne ST, Boyle P, Holford TR, Liu WL (1992). Epidemiology of non-Hodgkin lymphoma in Connecticut. 1935–1988.. Cancer.

[2] Jemal A, Siegel R, Ward E, Hao Y, Xu J (2008). Cancer statistics, 2008.. CA Cancer J Clin.

[3] Fisher SG, Fisher RI (2004). The epidemiology of non-Hodgkin's lymphoma.. Oncogene.

[4] Muller AM, Ihorst G, Mertelsmann R, Engelhardt M (2005). Epidemiology of non-Hodgkin's lymphoma (NHL): trends, geographic distribution, and etiology.. Ann Hematol.

[5] Morton LM, Wang SS, Devesa SS, Hartge P, Weisenburger DD (2006). Lymphoma incidence patterns by WHO subtype in the United States, 1992–2001.. Blood.

[6] Tanriverdi F, Silveira LF, MacColl GS, Bouloux PM (2003). The hypothalamic-pituitary-gonadal axis: immune function and autoimmunity.. J Endocrinol.

[7] Olsson H, Olsson ML, Ranstam J (1990). Late age at first full-term pregnancy as a risk factor for women with malignant lymphoma.. Neoplasma.

[8] Adami HO, Tsaih S, Lambe M, Hsieh C, Adami J (1997). Pregnancy and risk of non-Hodgkin's lymphoma: a prospective study.. Int J Cancer.

[9] Tavani A, Pregnolato A, La Vecchia C, Franceschi S (1997). A case-control study of reproductive factors and risk of lymphomas and myelomas.. Leuk Res.

[10] Nelson RA, Levine AM, Bernstein L (2001). Reproductive factors and risk of intermediate- or high-grade B-Cell non-Hodgkin's lymphoma in women.. J Clin Oncol.

[11] Zhang Y, Holford TR, Leaderer B, Boyle P, Zahm SH (2004). Menstrual and reproductive factors and risk of non-Hodgkin's lymphoma among Connecticut women.. Am J Epidemiol.

[12] Lee JS, Bracci PM, Holly EA (2008). Non-Hodgkin lymphoma in women: reproductive factors and exogenous hormone use.. Am J Epidemiol.

[13] Frisch M, Wohlfahrt J, Westergaard T, Hjalgrim H, Melbye M (1997). Induced abortion and the risk of non-Hodgkin's lymphoma.. Int J Cancer.

[14] Cerhan JR, Habermann TM, Vachon CM, Putnam SD, Zheng W (2002). Menstrual and reproductive factors and risk of non-Hodgkin lymphoma: the Iowa women's health study (United States).. Cancer Causes Control.

[15] Frisch M, Pedersen BV, Wohlfahrt J, Hjalgrim H, Biggar RJ (2006). Reproductive patterns and non-Hodgkin lymphoma risk in Danish women and men.. Eur J Epidemiol.

[16] Morton LM, Wang SS, Richesson DA, Schatzkin A, Hollenbeck AR (2009). Reproductive factors, exogenous hormone use and risk of lymphoid neoplasms among women in the National Institutes of Health-AARP Diet and Health Study Cohort.. Int J Cancer.

[17] Hennessy BT, Hanrahan EO, Daly PA (2004). Non-Hodgkin lymphoma: an update.. Lancet Oncol.

[18] Rosenberg S, Berard C, Brown B (1982). National Cancer Institute sponsored study of classifications of non-Hodgkin's lymphomas: summary and description of a working formulation for clinical usage.. Cancer.

[19] Harris NL, Jaffe ES, Stein H, Banks PM, Chan JK (1994). A revised European-American classification of lymphoid neoplasms: a proposal from the International Lymphoma Study Group.. Blood.

[20] Kleihues P, Sobin L, Jaffe E, Harris N, Stein H, Vardiman J (2001). Pathology and genetics of tumours of haematopoietic and lymphoid tissues..

[21] Morton LM, Turner JJ, Cerhan JR, Linet MS, Treseler PA (2007). Proposed classification of lymphoid neoplasms for epidemiologic research from the Pathology Working Group of the International Lymphoma Epidemiology Consortium (InterLymph).. Blood.

[22] Bernstein L, Allen M, Anton-Culver H, Deapen D, Horn-Ross PL (2002). High breast cancer incidence rates among California teachers: results from the California Teachers Study (United States).. Cancer Causes Control.

[23] Kwong SL, Allen M, Wright WE (December 2005). Cancer in California: 1988–2002..

[24] Reynolds P, Hurley S, Goldberg DE, Anton-Culver H, Bernstein L (2004). Regional variations in breast cancer among california teachers.. Epidemiology.

[25] Tulchinsky D, Hobel CJ, Yeager E, Marshall JR (1972). Plasma estrone, estradiol, estriol, progesterone, and 17-hydroxyprogesterone in human pregnancy. I. Normal pregnancy.. Am J Obstet Gynecol.

[26] Lindberg BS, Johansson ED, Nilsson BA (1974). Plasma levels of nonconjugated oestrone, oestradiol-17beta and oestriol during uncomplicated pregnancy.. Acta Obstet Gynecol Scand.

[27] MacMahon B, Trichopoulos D, Brown J, Andersen AP, Cole P (1982). Age at menarche, urine estrogens and breast cancer risk.. Int J Cancer.

[28] Vihko R, Apter D (1984). Endocrine characteristics of adolescent menstrual cycles: impact of early menarche.. J Steroid Biochem.

[29] Raghupathy R, Kalinka J (2008). Cytokine imbalance in pregnancy complications and its modulation.. Front Biosci.

[30] Moor RM (1968). Foetal homeostasis: conceptus-ovary endocrine balance.. Proc R Soc Med.

[31] Druckmann R, Druckmann MA (2005). Progesterone and the immunology of pregnancy.. J Steroid Biochem Mol Biol.

[32] Bernstein L, Ross RK (1992). Prior medication use and health history as risk factors for non-Hodgkin's lymphoma: preliminary results from a case-control study in Los Angeles County.. Cancer Res.

[33] Holly EA, Lele C, Bracci PM, McGrath MS (1999). Case-control study of non-Hodgkin's lymphoma among women and heterosexual men in the San Francisco Bay Area, California.. Am J Epidemiol.

